# Contrasting Experimental Rodent Aftercare With Human Clinical Treatment for Cervical Spinal Cord Injury: Bridging the Translational “Valley of Death”

**DOI:** 10.1089/neu.2023.0314

**Published:** 2023-11-30

**Authors:** Aaron L. Silverstein, Katelyn G. Lawson, H. Francis Farhadi, Warren J. Alilain

**Affiliations:** ^1^ Department of Neuroscience, Spinal Cord and Brain Injury Research Center, College of Medicine, University of Kentucky, Lexington, Kentucky, USA.; ^2^ Department of Neurosurgery, Spinal Cord and Brain Injury Research Center, College of Medicine, University of Kentucky, Lexington, Kentucky, USA.

**Keywords:** cervical spinal cord injury, clinical care, rodent models, spinal cord injury, translation

## Abstract

More than half of all spinal cord injuries (SCIs) occur at the cervical level and often lead to life-threatening breathing motor dysfunction. The C2 hemisection (C2Hx) and high cervical contusion mouse and rat models of SCI are widely utilized both to understand the pathological effects of SCI and to develop potential therapies. Despite rigorous research effort, pre-clinical therapeutics studied in those animal models of SCI sometimes fail when evaluated in the clinical setting. Differences between standard-of-care treatment for acute SCI administered to clinical populations and experimental animal models of SCI could influence the heterogeneity of outcome between pre-clinical and clinical studies. In this review, we have summarized both the standard clinical interventions used to treat patients with cervical SCI and the various veterinary aftercare protocols used to care for rats and mice after experimentally induced C2Hx and high cervical contusion models of SCI. Through this analysis, we have identified areas of marked dissimilarity between clinical and veterinary protocols and suggest the modification of pre-clinical animal care particularly with respect to analgesia, anticoagulative measures, and stress ulcer prophylaxis. In our discussion, we intend to inspire consideration of potential changes to aftercare for animal subjects of experimental SCI that may help to bridge the translational “Valley of Death” and ultimately contribute more effectively to finding treatments capable of restoring independent breathing function to persons with cervical SCI.

## Introduction

Almost 300,000 Americans currently live with spinal cord injuries (SCIs) ,and there are approximately 18,000 new injuries each year.^1^ Devastatingly, this might be an underestimate, because statistics from individuals who sustain an immediately life-ending injury are not fully counted.^1^ Among those who survive the acute phase of SCI, well over half have cervical level injuries and are at risk for profound, life-threatening respiratory dysfunction.^1,2^

Despite the serious impact that cervical SCI has on affected individuals' health and quality of life, there is no current clinical treatment capable of restoring respiratory function to pre-injury functionality. This deficit of available therapy is not for lack of research effort. Over the past 15 years, the National Institutes of Health (NIH) has devoted more than $83M to fund 255 separate projects that all relate to respiratory function after cervical-level SCIs.^3^ Across SCI research as a whole, NIH funding for a total of almost 10,000 projects has exceeded $3 billion over that same time period.^4^

In perspective, the funding for all NIH research grants totaled ∼$93.3 billion for those 15 years, meaning that cervical SCI/respiratory and SCI funding respectively constituted 0.09% and 3% of the scope of all NIH-supported research.^5^ While many of the therapies produced through this funding have been successful in pre-clinical models, these studies sometimes are not reproducible across different laboratories, and many fail when applied to the clinical setting. This points to a major issue in translating successful therapies to the clinic, an obstacle often described as the “Valley of Death.”

A compelling explanation for this phenomenon may lie in the variation in different laboratories' veterinary care of post-SCI animals and in the overall differences of these veterinary protocols with respect to current standard-of-care for clinical SCI. Treatment administered to clinical populations and animal models are often dissimilar and thus could differentially interact with experimental therapeutics, significantly influencing the heterogeneity of outcome between pre-clinical and clinical studies.

In fairness, it is important to recognize that veterinary aftercare standards have been designed primarily to provide the most efficient and cost-effective prevention of surgical complications for animals and not with the intention of recapitulating clinical care for humans. We suggest, however, that this review will illuminate some desirable modifications to pre-clinical aftercare protocols that will enhance the success of therapeutic translation from the bench to the bedside without sacrificing the necessary and valuable protections in place for ethical and humane use of experimental animals.

Thus, we will first describe the current standards of clinical treatment for acute cervical SCI in adults before we evaluate and compare with aftercare protocols from multiple laboratories utilizing the C2 hemisection and high cervical contusion models of SCI in mice and rats.

## Methods

### Review of clinical standard of care

In this review, our goal is first to summarize current standards of clinical care for adults with acute SCI in the human population. Thus, we referenced the most recent “Guidelines for the Management of Acute Cervical Spine and Spinal Cord Injuries” published by the Congress of Neurological Surgeons and the American Association of Neurological Surgeons as well as the clinical practice guideline entitled “Early Acute Management in Adults with Spinal Cord Injury” published in 2008 by the Consortium for Spinal Cord Medicine.^6–10^

In addition, we referenced UpToDate,^®^ an online database for clinical care standards described as an “Evidence-Based Clinical Decision Support System” offered by Wolters Kluwer. UpToDate^®^ is widely utilized by physicians and healthcare providers for point-of-care reference as they make clinical decisions and so substantively contributes to a summary of practices currently utilized to manage acute SCI.

We utilized UpToDate^®^ both to cite its peer-reviewed and edited content directly and also to find journal articles from its cited sources provided for each healthcare summary. The primary summaries cited were “Acute traumatic spinal cord injury,” “Anesthesia for adults with acute spinal cord injury,” and “Overview of inpatient management of the adult trauma patient.”^11–13^ We also referenced other UpToDate^®^ summaries concerning care for medically related complications as are reported completely in our Works Cited list.^2,14–20^

Finally, we searched PubMed^®^ for published, peer-reviewed journal articles describing healthcare guidelines for acute care of adults with SCI. We prioritized reference of the most recent guidelines, reviews, and meta-analyses available for a better understanding of current care standards. Our evaluation of PubMed^®^ results generated by our search queries was not intended to be exhaustive, but qualitative. Our collaborator and co-author, practicing spinal neurosurgeon, and researcher Dr. H. Francis Farhadi, provided oversight of our description of current standard of care for SCI.

### Review of high cervical SCI surgery veterinary aftercare

Our focus for this portion of the review was on commonly utilized rodent models of high cervical SCI defined as C4 and above. Thus, we conducted a systematic literature search in PubMed^®^ to find publications describing aftercare protocols for contusive and hemisection injuries in mice and rats. Search inputs were as follows: (C2 hemisection rat) OR (C2Hx rat) OR (C2 hemisection mouse) OR (C2 hemisection mice) OR (C2Hx mouse) OR (C2Hx mice) OR (high cervical contusion rat) OR (high cervical contusion mouse) OR (high cervical contusion mice). Literature reviews, clinical studies, and articles not written in English were excluded.

After closer evaluation of study content, articles were also excluded if they did not utilize a contusive or hemisection injury model, if the level of injury fell caudal to the C4 spinal segment, if the induced SCI was a terminal procedure, or if a description of aftercare methods was omitted. We analyzed at least five of the most recent qualifying publications from each senior author included in the initial list of publications compiled by our search. In cases wherein a particular senior author had fewer than five publications meeting our criteria, all were evaluated. Of the 192 entries returned by our initial search criteria, 98 were ultimately included in this review.

### Clinical care for spinal cord injury

The first recorded description of medical treatment of spinal cord injury can be found in the Edwin Smith-translated surgical papyrus thought to be written by the Egyptian physician Imhotep in the 30th century B.C.^21^ The writer described SCI as “an ailment not to be treated,” and such a bleak assessment of SCI prognosis remained in essence unchanged for almost 5000 years until the 19th century A.D.^21,22^ Once medical professionals began to propose and develop treatments for persons with SCIs, their efforts targeted prevention of additional central neurotrauma after the initial insult through surgical decompression and, peripherally, on managing or preventing complications.^22^

Even now, promising pre-clinical and clinical therapies targeted at the reversal of functional loss induced by SCI have not yet become a part of standard clinical practice.^23^ Today, the standard of care still consists exclusively of treatments that seek to preserve remaining neurological function and prevent complications, but these are now better defined and standardized according to the history of injury as well as clinical signs and symptoms. These standards of care can be categorized and discussed as below with findings summarized in Table 1.

**Table 1.** Summary of Clinical Care Standards for High Cervical SCI

Intervention	Purpose	Clinical Care
Airway & breathing	Maintenance of respiratory function	Intubate at first indication of respiratory dysfunction, ventilating with 10 mL/kg body mass & PEEP of 0-5 cmH_2_O.
Anesthesia for intubation	Facilitation of intubation	Propofol & thiopental or etomidate & ketamine with neuromuscular blocker.
Chest physical therapy	Decrease retention of lung secretions and atelectasis	As soon as tolerated, training to increase breathing ability and maintenance of respiratory health.
Oral hygiene care	Decrease infection from aspirated mouth flora	Assisted toothbrushing at least once per day, often with paste containing chlorhexidine.
Cardiovascular management	Maintenance of adequate cardiac output and perfusion	Correction of active bleeding, early infusion of I.V. fluids and/or blood, and norepinephrine administration to maintain MAP of 85-90 mmHg.
Analgesics	Pain relief	Opioids & acetaminophen, no perioperative NSAIDs, and generally rare NSAID use.
Antibiotics	Infection treatment or prophylaxis	Prophylaxis not indicated for SCI itself, but various antibiotics used for SCI complications and prophylaxis for surgery and penetrating trauma.
Anticoagulation	Prevention of coagulative complications	Treatment with subcutaneous LMWH as soon as possible post-SCI and for at least 12 weeks, briefly interrupted for surgery.
Dermatologic care	Avoidance of pressure sore-formation	Special bedding, positional shifting with frequent skin integrity inspection.
Nutrition	Adequate nutrition	Early enteral feeding if swallow function allows, monitor/correct glycemia.
Stress ulcer prophylaxis	Prevent gastroduodenal stress ulcers	Prophylaxis with proton pump inhibitors for 4 wks.
Temperature regulation	Avert hypothermia and hyperthermia	Warm with blanketing & warmed fluids, adjust to prevent overheating.
Timing of spinal surgery	Prevention of further spinal cord damage	Surgical stabilization, reduction, or decompression within 24 hrs. post-SCI as medically feasible.
Anesthesia for spinal surgery	Facilitation of surgery	Closed reduction: Diazepam or meperidine Induction for surgery with general anesthesia: I.V. propofol, ketamine, and etomidate, not inhalables. Maintenance: For somatosensory evoked potentials I.V. propofol, inhaled sevoflurane, and I.V. opioid (remifentanil, sufentanil, or fentanyl) with any neuromuscular blocking agent. Motor evoked potentials (MEP) or electromyography (EMG) no neuromuscular blocking agents For MEP: propofol, sevoflurane, and an opioid For EMG: sevoflurane and an opioid Electroencephalogram or electrocorticography I.V. remifentanil and a muscle relaxant with either inhaled anesthetic (nitrous oxide or sevoflurane) or I.V. dexmedetomidine and propofol.
Urinary and bowel care	Bladder voiding and defecation	Urinary catheterization and bowel assistance as needed for prevention of bladder distension and ≥1 bowel movement per day.
Steroids	Neuroprotection	Methylprednisolone not recommended as first-line SCI treatment.

#### Airway and breathing interventions

Respiratory failure is common after cervical or high thoracic SCI as signified by increasing respiratory rate, decreasing forced vital capacity, increasing pCO2, or decreasing PO2 and necessitates urgent intubation and mechanical ventilation.^11^ Special care should be taken in cases of cervical immobilization.^11^ Even in patients who do not require mechanical ventilation, hypoxemia is common and must be recognized and addressed via administration of supplemental oxygen.^8^ Because of the common need for intensive cardiopulmonary monitoring and ventilation, the intensive care unit (ICU) is the most appropriate hospital setting for cervical SCI care.^6,8^

It is best to perform intubation for ventilation in controlled, non-emergent circumstances, so it is preferred to intubate at the first signs of respiratory distress in persons with SCIs at C5 or above.^8,24^ For patients likely to require long-term ventilation, tracheostomy is recommended as an early intervention, and orotracheal is preferred to nasotracheal intubation in the short-term.^24^ For the most part, ventilator settings are left to the judgment of the attending physician, but it is important to balance the concerns regarding ventilatory-induced lung injury at higher tidal volumes and increased collapse of small airways (atelectasis) at lower volumes.^14^ As a guide, care standards suggest a tidal volume of 10 mL/kg of body mass.^14^

Positive end-expiratory pressure (PEEP) consists of residual positive pressure at the end of each inspiration imposed by mechanical ventilation to prevent atelectasis because the inflation generated by positive pressure ventilation decreases with each expiration.^14,25^ Optimal settings in patients with SCI who have respiratory failure are not fully standardized, but typical practice is to use 0-5 cm H2O of PEEP, avoiding overinflation.^14^ Exogenous surfactant administration also can serve to prevent atelectasis.^25^ Even in spontaneously breathing patients, positive pressure is often administered through nasal cannula to enhance airway opening.

##### Anesthesia for intubation

For facilitation of intubation induction of anesthesia via propofol and thiopental is common in patients who have no hemodynamic instability, but etomidate and ketamine are viable alternatives and are likely safer for patients at risk for hypotension.^6^ If within 48 h of injury, a short-acting neuromuscular blocker such as succinylcholine should be used, but after 48h a nondepolarizing neuromuscular blocking agent is the paralytic of choice.^6^

##### Chest physical therapy for patients with respiratory motor dysfunction

Beginning as soon as possible after injury, all persons with cervical or thoracic SCI are recommended to undergo chest physical therapy whether intubated or not to assist with improving respiratory function and health.^6,14^ Techniques employed include deep breathing, glossopharyngeal breathing, and accessory muscle training, positioning changes, postural secretion drainage, manually assisted (quad) coughing, percussion, vibration, and mechanical insufflation-exsufflation.^6,14^ Suctioning is also performed, through the tracheostomy if present or via the nasotracheal or orotracheal route.^6,14^ Suctioning in non-intubated patients, however, misses the left mainstem bronchus 90% of the time because of anatomical orientation, and is also uncomfortable for the patient and could damage the larynx.^14^

##### Oral hygiene care for mechanically ventilated patients

Good oral care significantly reduces the rate of ventilator-associated pneumonia by reducing the prevalence of mouth flora that could be aspirated while the epiglottis is bypassed by an endotracheal tube.^26,27^ The specific care protocol is not standardized, however, with different sources indicating different frequencies of tooth brushing and varied application methods for the various types of antimicrobial paste used.^26,27^ In the ICU, nurses typically perform their hospital's oral care procedures at least once per day, often including chlorhexidine application.^26,27^

#### Cardiovascular management

After a high traumatic SCI, there is great risk of hemodynamic and/or neurogenic shock stemming from blood loss or interruption of sympathetic tone, respectively.^8,11^ Shock manifests as inadequate cardiac output and/or hypotension that worsen neurological outcome and can lead to death by hypoperfusion of vital tissues.^8,11,28^ Many post-SCI patients able to breathe without mechanical ventilation still experience hypoxemia despite adequate alveolar ventilation likely because of ventilation/perfusion mismatch.^8^ Thus, the ICU is the most appropriate care setting for patients with SCI because of the close monitoring necessary to identify and manage cardiopulmonary issues.^6,8^

Early infusion of intravenous fluids and/or blood transfusions are vital to maintain an appropriate mean arterial blood pressure (BP), which is considered to be 85–90 mm Hg by most guidelines.^6,8,11,28,29^ Other therapeutic targets include systolic blood pressure of 90–100 mm Hg and heart rate (HR) of 60–100 beats per minute.^11,29^ Fluid status must be monitored (target of urine output >30 mL/h) to avoid exacerbation of spinal cord swelling, kidney dysfunction, and edema, as well as other relevant pathologies.^11,29^ In addition to fluid resuscitation, sources of cardiovascular dysfunction apart from neurogenic shock (e.g., blood loss) should be determined and remedied.

Cervical or high thoracic SCIs (above T6) often lead to loss of sympathetic innervation and might require treatment with norepinephrine for its chronotropic and vasopressor activity at both alpha- and beta-adrenergic receptors.^6,28,29^ Vasopressors with only alpha-adrenergic activity such as phenylephrine or midrodine are more appropriate for management of hypotension caused by more distal SCIs, but caution should be used with midrodine because of its propensity to cause urinary retention.^6,28,29^ Close monitoring is required to avoid inducing elevated intracerebral pressure and cerebral edema via vasopressor treatment.^28^

Bradycardia might require management with external pacing, atropine, or other pacemaker drugs,6,11 During anesthesia, ephedrine is preferred because of its alpha and beta-1 agonist activity that increases BP and cardiac output in persons with bradycardia.^18^ Norepinephrine and epinephrine are alternatives, but either requires central line placement for administration or is more suited for acute hypotensive episodes such as cardiac arrest or anaphylaxis, respectively.^18^ Phenylephrine or vasopressin/terlipressin can be used during anesthesia in persons with elevated or normal HR, but vasopressin and its analogues are most appropriate for refractory hypotension and are used as a last resort in context of neurological injury because of their propensity to induce cerebral vasoconstriction.^18^

#### Analgesics

Analgesia is important to ameliorate individuals' pain after SCI. Ideally, the chosen analgesic will have fast onset, no risk of causing dependence, allodynia, or hyperalgesia, and have no side effects.^19^ All of these criteria are not to be met in any single agent, so a multi-modal approach is usually taken.^19^ Overall, intravenous administration is preferred acutely after SCI. Opioids are the first-line treatment for pain in the critically ill patient, but their use after SCI, especially at the cervical level, might be limited because of their respiratory depressive effects because patients' respiratory function is often impaired after injury.^19^

In addition, opioids' sedative effects tend to impair neuromonitoring, which is often necessary acutely after SCI for diagnostic and surgical purposes.^6,11,17^ For these patients, a short acting opioid is most appropriate, such as fentanyl or remifentanil.^19^ Morphine is also commonly used, but has increased proclivity toward causing pruritis and has a longer duration of action.

For non-opioid analgesia, acetaminophen is most commonly utilized, while non-steroidal anti-inflammatory drugs (NSAIDs) such as ibuprofen are uncommonly used.^19^ The primary contraindication to acetaminophen is hepatic insufficiency while the list of contraindications to NSAIDs is much more extensive, including recent surgery and surgical bleeding, non-surgical bleeding, renal dysfunction, platelet abnormality, heart failure, cirrhosis, asthma, and angiotensin-converting enzyme (ACE) inhibitor therapy.^19^

Further, cell culture and animal studies have indicated that NSAID use can inhibit bone healing—a risk worth considering for human cases of SCI, despite some conflicting clinical data discussed in recent meta-analyses.^30–32^ Patients with SCI often undergo surgery, need unimpaired bone regrowth, and are likely to have other comorbidities that contraindicate NSAID therapy, rendering acetaminophen a more appropriate analgesic.^19^

For refractory pain, ketamine has shown promise and can reduce the necessary opioid dosage to attain an appropriate level of pain control.^6,19^ Gabapentinoids such as gabapentin or pregabalin are additional options available for management of specifically neuropathic pain, or to reduce dependence on other analgesics in the long term.^6,19^ For persons with SCI unable to control their own analgesic administration via manipulation of a button, breath controlled methods can be used.^6^

#### Antibiotics

More rostral SCIs confer greater risk of pneumonia than more caudal injuries.^33–35^ In addition, SCI in general renders patients more susceptible to infections of various sorts, a phenomenon thought to be influenced by a neurogenic mechanism termed spinal cord injury-induced immune deficiency syndrome (SCI-IDS).^33–35^ In addition to pneumonia, other common infectious pathologies associated with cervical SCI include urinary tract infections (UTIs) and infections at the site of a penetrating injury.^28^

The use of antibiotics is not indicated as prophylaxis for infection related to SCI per se because of guidelines intended to minimize antibiotic overuse and antibiotic resistance.^26^ Prophylaxis can become necessary, however, if surgery is needed, or if there is polytrauma involving open fractures or penetrating injuries to the thorax.^13^ When antibiotic prophylaxis is warranted or when management of infection from a penetrating injury is necessary, the empiric antibiotic therapy is highly dependent on the specific context and anatomical location of injury or surgery type.

Empiric therapy for hospital or ventilator-associated pneumonia should cover *Staphylococcus aureus, Pseudomonas aeruginosa*, and other gram-negative bacilli while the specific antibiotics utilized depend on hospital-specific prevalence of bacterial strains and patient characteristics.^16^ For UTIs in patients with SCI treated within the ICU, recommended empiric therapy should be instituted with both an antipseudomonal carbapenem and either vancomycin or a combination of both linezolid and daptomycin to cover methicillin-resistant *S. aureus* (MRSA).^15^ For all empiric regimens, treatments should appropriately change as analysis of patient samples and hospital-specific characteristics dictate.

#### Anticoagulation

Persons with SCI are designated as a population at high risk for blood clot formation because immobilization after SCI often causes venous stasis leading to hypercoagulability and possibly life-threatening complications such as deep vein thrombosis and pulmonary embolism.^29^ Thus, anticoagulative prophylaxis with subcutaneous low-molecular-weight heparin (LMWH) is the standard of care after SCI.

The LMWH is usually accompanied by mechanical prophylaxis that includes use of compression stockings and intermittent pneumatic compression.^14,29^ In cases wherein there is a heightened bleeding risk, mechanical measures can be used without pharmacological intervention. It is recommended to implement LMWH therapy as soon as possible after SCI and for at least 12 weeks, with brief interruption for necessary surgery.^14,29^ Unfractionated heparin is an alternative if LMWH cannot be used, but evidence indicates lower efficacy.^14^ Treatment with warfarin or direct anticoagulants is not recommended for acute SCI because of the heightened bleeding risk.^14^

#### Dermatologic care

Measures to prevent pressure sore formation are required for every immobilized patient with SCI and especially those with loss of somatosensation. Once patients' injuries are stabilized, logrolling is the standard technique utilized to shift the patient such that one aspect of the body is not constantly weight bearing.^6,11^ Use of pressure-reducing cushions and bedding is also effective. Alternatively, a specialized rotating bed for patients with SCI can be used where available.^11^ Frequent inspection of the skin integrity, especially at dependent areas overlying bony prominences, is vital as well as elimination of excess moisture and heat applied near the skin.^6^

#### Nutrition

Swallow function must be evaluated before beginning oral feeding in any patient with cervical SCI.^6^ Only if risk of aspiration is low can oral feeding safely occur. Enteral feeding is preferred above parenteral whenever possible and should begin early, ideally within 72h of injury, once the patient is stable and able to tolerate it.^6,7,9,13^ Contraindications to enteral nutrition include progressive ileus, bowel obstruction, massive bowel resection, malabsorption, splanchnic hypoperfusion, fistulae, and insufficiency of enteral nutrition to meet caloric requirements.^7,13^ Total caloric requirements should be calculated by a 30-min energy expenditure measurement via indirect calorimetry, although it is important to correct for its propensity for overestimation.^6,7,9^

Blood glucose levels should be monitored and treatment instituted to prevent hyperglycemia. Hyperglycemia in patients with SCI is common and is associated with greater death and disability at one year after injury.^6,13,36,37^ The standard for control of hyperglycemia is intensive insulin therapy, with a target range for blood glucose of 80–110 mg/dL.^6,13,36^ Hypoglycemia is best prevented through nutrition according to guidelines described above to prevent exacerbation of neurological injury.

#### Stress ulcer prophylaxis

Current standards define traumatic SCI patients and those on mechanical ventilation for >48h as high risk for stress ulcers and ulcer-associated gastrointestinal bleeding and thus recommend that all such patients receive prophylaxis for 4 weeks.^6,11,13,20,38^ Proton pump inhibitors (PPIs) are more protective against gastrointestinal bleeding than histamine receptor 2 (H2) blockers, and pantoprazole is the most common enterally administered PPI prescribed.^6,11,20^ There is an increased risk of *Clostridioides difficile* colitis, however, associated with PPI prophylaxis, that could recommend prescription of H2 blockers as a possible alternative.^6,13,20^

#### Temperature regulation

Poikilothermia, or an inability to maintain core body temperature, is common in cervical and high thoracic SCI and especially during anesthesia.^6,12^ Thus, body temperature must be measured and regulated appropriately until the individual is able to maintain appropriate body temperature ∼37°C.^6,12^ Hypothermia is the most common manifestation of temperature dysfunction in persons with SCI, and so blanketing (heated or not) and warming of intravenous fluids are common and effective interventions.^12^ Continued monitoring is important to avoid accidental hyperthermia, because sweating function is often impaired below spinal cord lesion.

#### Timing of spinal surgery

For penetrating SCIs, surgery is often indicated for foreign body removal and disinfection.^11^ To prevent neurological deterioration and achieve mechanical stability of the spine, surgical stabilization, reduction, or decompression is indicated if spinal instability, ligamentous damage, or neurological dysfunction is severe or progressive enough.^6,11,29^

For cervical SCI, these metrics can be assessed through the Subaxial Cervical Spine Injury Classification (SLIC) scales determined by analysis of imaging and clinical findings wherein a score >4 indicates a need for operative management.^29^ A T2 MRI is the gold standard for evaluation of spinal cord compression and vascular or ligamentous injury when available and clinically appropriate, while CT is the first-line imaging modality but cannot image spinal cord or other soft tissue as well as MRI.^11,25,29^

Closed reduction is a management option for cervical spine fractures with subluxation, but not for thoracic or lumbar injuries.^11^ Irrespective of the neural complete or incomplete nature of the SCI as assessed by the American Spinal Injury Association Impairment Scale (AIS), current evidence supports early neural decompression within 24h of SCI when medically feasible.^6,11,25,29,39^

##### Anesthesia for spinal surgery

For facilitation of spinal surgery, there are particular considerations with respect to appropriate anesthesia. Closed reduction can be facilitated through administration of a muscle relaxant or analgesic such as diazepam or meperidine. Induction of general anesthesia for open surgical approaches is intravenous, commonly consisting of propofol, ketamine, and etomidate.^12^ Of note, inhalable anesthesia is not commonly utilized for induction.^12^

Maintenance protocols for general anesthesia differ according to the type of neuromonitoring that is performed during the procedure.^12,17^ All inhaled agents tend to affect neuromonitoring to a greater degree than intravenous drugs, but many neuromonitoring modalities can tolerate low-dose sevoflurane.^6,17^ For cases indicating measurement of somatosensory evoked potentials, an appropriate maintenance protocol consists of intravenous propofol, inhaled sevoflurane, and intravenous opioid (remifentanil, sufentanil, or fentanyl) with any neuromuscular blocking agent (succinylcholine, rocuronium).^6,17^

No neuromuscular blocking agents can be used during motor evoked potentials (MEP) or electromyography (EMG).^17^ Specific protocols for MEP consist of propofol, sevoflurane, and an opioid while EMG requires low-dose sevoflurane and an opioid.^17^ Testing with an electroencephalogram or electrocorticography indicates intravenous remifentanil and a muscle relaxant with either inhaled anesthetic (nitrous oxide or sevoflurane) or intravenous dexmedetomidine and propofol.^17^

#### Urinary and bowel care

Urinary dysfunction is common acutely after SCI, and indwelling urinary catheterization is part of the initial patient assessment unless contraindicated or in cases wherein there is no suspicion or sign of urinary retention.^6,11^ Bladder distention can be assessed by palpation or ultrasound.^6,11^ After 3 or 4 days post-injury, indwelling catheters can sometimes safely be removed in preference for intermittent catheterization to decrease infection risk.^6,11^ For persons with SCI and gut dysmotility, one bowel movement per day is the goal.^6^

#### Steroids

Methylprednisolone (MP) was observed to have efficacy in reducing peritumoral cerebral edema and inspired MP's off-label use in the clinic to reduce edema in SCI beginning in the 1960s.^40^ Promisingly, animal experiments demonstrated that MP treatment reduced edema, prevented intracellular hypokalemia, and improved neurological recovery after SCI because of its inhibition of lipid peroxidation and other mechanisms.^11,40^ Multiple randomized controlled trials in the human population, however, have since evaluated MP after SCI, but any neurological improvement attributed to MP treatment has tended to be small and inconsistent while adverse effects attributable to MP were more robust and repeatable.^6,9,11,41^ Thus, MP is no longer recommended as a first-line treatment for SCI.^6,9,11,41^ If used despite recommendations, the best MP protocol for SCI seems to begin within 8h of injury and last for no more than 24h.^25^

### Veterinary care standards after C2 hemisection and high cervical contusion injury in mice and rats

The C2 hemisection (C2Hx) is a well-established animal model of a controlled, laceration-type incomplete SCI that generates consistent respiratory motor deficits ipsilateral to the lesion.^42–46^ While the C2Hx is vital for the study of treatments to ameliorate a controlled, unilateral functional deficit, cervical contusion utilizes a clinically relevant forceful impact to model SCI.^47–50^ High cervical spinal cord contusions, however, lead to less consistently severe respiratory motor deficits than hemisections.^47–50^

Because of these two injury models' complementary strengths and weaknesses, studies using C2Hxs and cervical contusions together portray a more complete picture of high cervical SCI than either approach alone. Thus, both experimental models in mice and rats are vital to our comparison of veterinary care protocols to the clinical treatment of spontaneous SCI in humans.

In the following sections, we will summarize veterinary care protocols used by the various laboratories studying C2Hx and high cervical contusions in mice and rats, organizing (where applicable) each intervention into the same categories we used to describe clinical care standard for SCI (Table 2). Where protocols substantively differ between various laboratories, we will list the specific interventions used by each research group.

**Table 2.** Summary of Veterinary Care for Experimental High Cervical SCI in Murine Animals

Intervention	Purpose	Clinical Care
Airway & breathing	Intraoperative blood gas control	Some laboratories intubate and ventilate animals during SCI surgery.
Cardiovascular management	Replace blood loss	Fluid injections during and after surgery.
Analgesics	Pain relief	Most common:. Opioid or opioid+NSAID.
		Less common: NSAID alone, opioid+ acetaminophen, or non-NSAID COX-3 modulators.
Antibiotics	Infection treatment or prophylaxis	Most common: No prophylactic antibiotics.
		Less common: Systemic prophylactic treatment with various agents.
Anticoagulation	Prevention of coagulative complications	None
Dermatologic care	Avoidance of skin damage	Surgical site care and regular assessment.
Nutrition	Adequate nutrition	Most common: Diet supplementation & weight monitoring.
		Less common: Dextrose or glucose infusion.
Stress ulcer prophylaxis	Prevent gastroduodenal stress ulcers	None
Temperature regulation	Avert hypothermia & hyperthermia	Most common: Volitional access to heating pad.
Anesthesia for spinal surgery	Facilitation of surgery to induce C2Hx or high cervical conusion	Most common: Xylazine(or medetomidine) cocktail with ketamine.
		Less common: Inhalable isoflurane or halothane, pentobarbital, chloral hydrate, or fentanyl and midazolam.
Urinary and bowel care	Bladder voiding and defecation	Monitoring, bladder expression & bowel evacuation when needed.

#### Airway and breathing interventions

The C2Hx model of cervical SCI is designed intentionally to induce a unilateral respiratory deficit while leaving the opposite side unimpaired.^42–46^ Thus, animal subjects that receive a C2Hx surgery do not require intubation or mechanical ventilation for post-surgical survival.

The Bolser, Mitchell, Lukáčová, and Vinit laboratories, however, sometimes utilize intubation and mechanical ventilation to administer inhalable anesthetics during C2Hx surgeries, and those interventions become necessary for survival when animals experience respiratory arrest after a cervical contusion injury.^51–64^ In such cases, the same anesthetic agents are maintained that were in effect during the injury surgery. No additional considerations of PEEP, chest physical therapy, or oral hygiene are described for either intraoperative or post-injury intubation or mechanical ventilation.

#### Cardiovascular management

After SCI surgery, subcutaneous fluids (physiological saline or lactated Ringer solution) are often administered to maintain hydration.^43,47,48,51,54,55,59,65–106^ Murine subjects are usually provided access *ad libitum* to drinking water immediately after surgeries with accommodations for limited mobility such as extended waterspouts.

#### Analgesics

The Askew, Baekey, Bonay, Chang, Fehlings, Gonzalez-Rothi, Goshgarian, Hsu, Lane, Lee, Lepore, Mansart, Tsai, and sometimes the Alilain, Decherchi, Fuller, Nantwi, Reier, and Vinit laboratories implement post-SCI systemic analgesic monotherapy with buprenorphine (a partial mu opioid receptor agonist).^47,48,51,69–71,74–77,80–83,86,90,92-96,99–101,106–113^ The Gauthier laboratory used pentazocine (an opioid), the Lukáčová laboratory utilized metamizole (a non-opioid analgesic with activity at central cyclooxygenase-3), and the Novikov laboratory utilized flunixin (an NSAID) as monotherapy.^60,91,114,115^ For the Descherchi laboratory, their aftercare protocol sometimes consists of a combination of dextropropoxyphene (an opioid) and acetaminophen administered through drinking water.^87^ Post-SCI analgesic treatment for Kastner laboratory animals included acetaminophen with codeine and/or morphine sulfate.^84,116–118^

Drs. Sieck and Mantilla usually post-operatively treat their experimental rats and mice with either acetaminophen and buprenorphine together or acetaminophen alone.^119–128^ In one rat contusion study, the Sieck and Mantilla group administered the NSAID carprofen *ad libitum* through the drinking water in addition to injectable buprenorphine.^79^ In other studies, the Alilain, Bolser, Dale, Fuller, Mitchell, Mateika, Reier, Sankari, and Vinit laboratories utilize a dual systemic approach consisting of both buprenorphine and NSAID administration (either carprofen or meloxicam).^43,5256,58,59,61–65,67,68,73,78,88,89,97,103,129–133^

During and immediately following Alilain and Kastner laboratory SCI surgeries, bupivacaine or procaine (sodium channel blockers) are often used for short-term, localized paralytic and analgesic effects.^47,48,84,132^ The recent cervical hemi-contusion publication from the Fernández-Muñoz laboratory stated that they treated animals with analgesics, but the specific protocol was not detailed.^104^ Notably, the publications from the Cox, Feron, and Xu groups included no analgesic protocol for their post-SCI animals.^98,102,105^

#### Antibiotics

No systemic prophylactic or responsive antibiotic treatment was included in the cervical SCI care protocols described by the Alilain, Askew, Baekey, Bolser, Chang, Dale, Fehlings, Gonzalez-Rothi, Hsu, Lane, Lee, Mateika, Nantwi, Reier, Sankari, Tsai, or Xu laboratories, or in the majority of Lepore, Fuller, and Goshgarian laboratory publications. Some Fuller and Goshgarian laboratory articles, however, do include enrofloxacin treatment after surgery.^72,78^ One Lepore lab publication induced their C2Hx injury in immunosuppressed rats and thus included cephazolin in their post-operative treatment regimen.^81^ In addition, the Alilain laboratory applies topical triple antibiotic ointment to subjects' minor skin wounds as they arise, according to veterinary staff instruction (personal communication).

In their past experimentation, Drs. Sieck and Mantilla have pre-emptively treated their post-SCI animals with penicillin G, the Descherchi laboratory has used amoxicillin, the Lukáčová group used amoxicillin-clavulanic acid, the Mitchell group has often used enrofloxacin, and the Novikov laboratory has used benzylpenicillin for infection prophylaxis.^52–56,58–60,79,86,91,120,125,130,131^ The Feron and Kastner laboratories post-operatively treat animals with oxytetracycline and the Bonay, Mansart, and Vinit groups used trimethoprim and sulfadoxine.^64,84,86,98,111–113,116–118^

The Cox laboratory treated animals with gentamicin, although because their study was designed to investigate the neuroprotective effect of MP treatment, their prophylactic antibiotic treatment might have been motivated more to combat the risks of MP-induced immunosuppression than as part of standard post-SCI aftercare.^105^ Although the specific agent(s) were not mentioned, the Fernández-Muñoz laboratory described that they implemented responsive antibiotic therapy if their post-surgery rats showed signs of infection.^104^

#### Dermatological care

During cervical SCI surgery, ointment is applied to the anesthetized animals' eyes to prevent ocular damage.^67,132^ Other skincare provided to experimental animals across all literature surveyed was limited to preparation of the sites for aseptic or sterile procedure, closure of the surgical incision, nail trimming, monitoring and prevention of autophagy, and cleansing of the animals' surgical site, fur, eyes, and mouth as needed post-surgery.^42,43,47,48,51,53,56–74,77–84,86–97,99–104,106,107,109-113,116–121,123,129,132–135^ Experimenters did not employ any treatment as prophylaxis for pressure sores.

#### Nutrition

Murine subjects are usually provided access *ad libitum* to normal chow immediately after surgeries with accommodations for limited mobility such as food pellets placed on the cage floor. Animals' weight is monitored and diet supplemented with salient food choices at least until volitional feeding and normal defecation resumes.^43,53,54,57,66,67,71–74,77–79,83,84,86,92–97,99–102,104,106,108–110,133^ The Kastner laboratory includes parenteral glucose as standard post-operative treatment, and in one contusion publication the Mantilla and Sieck groups treated subjects with intravenous dextrose.^79,84,116–118^

#### Temperature regulation

During spinal injury surgeries, murine subjects usually are placed on a heating pad to maintain body temperature^54–56,67,68,71–73,77,78,85,97,106,111^ After surgery, animals are often allowed to recover with access to heat for at least a short time.^43,47,48,55,70,72,73,78,79,81–83,85–87,106,107,109,114,119,121,123^

#### Anesthesia for SCI surgery

All murine subjects discussed in this review received an experimentally induced SCI and thus underwent anesthesia to facilitate the surgery. For many of their studies, laboratories led by Drs. Alilain, Chang, Cox, Fernández-Muñoz, Fuller, Gonzalez-Rothi, Goshgarian, Gonzalez-Rothi, Hsu, Lane, Lee, Lepore, Mantilla, Mateika, Nantwi, Novikov, Reier, Sieck, Tsai, and Xu utilized an injectable cocktail of xylazine and ketamine for anesthesia and the Alilain, Chang, Gonzalez-Rothi, Hsu, Lane, Lee, Lepore, Reier, Tsai, and Xu laboratories further often apply a reversal agent for xylazine (yohimbine or atipamezole) after procedure completion^42,43,47,48,51,66,69–72,76,78–83,90–96,100–102,104,105,108–110,119–128,133,136–139^

The Lepore laboratory sometimes adds a dopamine antagonist (acepromazine) to their xylazine/ketamine cocktail for its properties as a muscle relaxant and sedative.^80,81,138^ The Roman laboratory utilized pentobarbital and the Kastner group utilized either pentobarbital, medetomidine/ketamine, or xylazine/ketamine injections for induction and maintenance of anesthesia and administered acetaminophen with codeine and/or morphine before surgery for sedative and analgesic effect.^84,116–118,140^

In some studies, the Goshgarian, Kastner, and Nantwi laboratories also pre-operatively used atropine sulfate to decrease airway secretions and promote adequate hemostasis.^84,108,109,136^ In some studies, the Fuller group induced and maintained anesthesia exclusively with inhalable isoflurane instead of injectables, and the Askew, Baekey, Bolser, Bonay, Dale, Decherchi, Fehlings, Mansart, Mitchell, Reier, Sankari, and Vinit laboratories followed a similar protocol for many of their SCI surgeries.^53–55,58,59,61–65,67,68,73–75,77,85,86,88,89,97,99,103,106,111–113,129^ In their one publication studying the C2/3 hemisection, the Lukáčová laboratory anesthetized rats solely with halothane.^60^

In some experiments, the Bolser, Mitchell, and Vinit laboratories injected animals with medetomidine or dexmedetomidine before isoflurane induction and used atipamezole to reverse the medetomidine once the C2Hx surgery was complete.^52,58,59,61–64^ In some of their work, the Decherchi, Feron, and Gauthier groups utilized injectable chloral hydrate as the sole anesthetic, and in one publication, the Feron and Gauthier laboratories added atropine to their regimen.^87,98,114,134^ The Ishii laboratory anesthetized their rats with both midazolam and fentanyl injections before C2Hx.^135^ For mouse studies, the Alilain and Lane laboratories induced and maintained anesthesia on isoflurane alone.^107,132^

#### Urinary and bowel care

While no consistently severe or long-lasting bladder or bowel impairment commonly results from models of incomplete cervical SCI in rats and mice, some research groups such as the Alilain, Cox, Decherchi, Goshgarian, Lee, Mitchell, and Sankari laboratories have included urinary and bowel monitoring and/or care as part of their post-surgical animal care.^47,48,72,73,86,87,95,97,105,109^ The Alilain, Decherchi, Lee, Mitchell, and Sankari laboratories performed checks daily and manually expressed bladders when needed until subjects became capable of spontaneous voiding (usually within a week).^47,48,73,86,87,95,97,105^ In the Goshgarian laboratory, urinary and gastrointestinal aftercare included manual urinary voiding and bowel evacuation while rats were still anesthetized to prevent urinary retention or bowel impaction.^72,109^ In those studies, bladder and bowel function were evaluated in the days after surgery and returned to normal within 2 days or less.^72,109^

#### Anticoagulation and stress ulcer prophylaxis

No published study of high cervical SCI includes any prophylactic or responsive anticoagulative intervention or stress ulcer treatment as part of standard aftercare for murine animals.

#### Steroids

Just as MP therapy is excluded from the current standard of care for clinical SCI, it is excluded from veterinary aftercare protocols for experimentally induced cervical SCI in murine animals.

## Discussion

Through our review of SCI care protocols used both in the clinic and in the laboratory, it is apparent that stark differences exist in two spheres. First, clinical care for spontaneously occurring SCIs in humans differs greatly from veterinary care given to animals with experimentally induced high cervical SCIs. It is important to acknowledge, however, that certain aspects of clinical care are not directly applicable to treatment of experimental animals. Second, the specifics of care delivered to mice and rats varies significantly between laboratories and even within laboratories from publication to publication. For perspective, the passage of time, governmental regulation, and even drug or equipment availability between institutions all differentially affect laboratories in their implementation of veterinary care.

Through our brief discussion we will describe which aspects of clinical care we assert to be most applicable to rodent models of SCI and advocate for specific modifications to veterinary care protocols to be instituted across laboratories based on our assessment of clinical practice.

### Utilizing clinical protocols to update post-SCI animal care

In the following section, we will systematically discuss the applicability of each clinical intervention to post-SCI veterinary aftercare protocols, compare and contrast veterinary and clinical care standards where both exist, and finally suggest amendments to animal care protocols for the field of cervical SCI research to evaluate and implement. Recognizing that the cost/benefit analysis of each additional change to aftercare procedures might not favor implementation for all research groups, we have organized our discussion in descending order of the importance that we attach to each suggested change to current post-SCI veterinary care protocols. Table 3 depicts the comparison of clinical and veterinary care currently in practice by the field and our summarized suggestions for improving the clinical relevance of murine studies by adapting animal aftercare to clinical practice, where practical.

**Table 3.** Comparison of Clinical and Veterinary Care for SCI and Suggestions for Modifications to Experimental Animal Aftercare

Intervention	Clinical Care	Current Veterinary Aftercare	Suggested Amendments to Veterinary SCI Aftercare
**High Priority**			
Analgesics	Opioids and acetaminophen, not NSAIDs.	Opioid or opioid+NSAID.	Acetaminophen + buprenorphine instead of NSAID use.
Anticoagulation	Treatment with subcutaneous LMWH within 72 hours post-SCI and for at least 12 weeks.	None	LMWH acutely after SCI and continued until chronic timepoint.
Stress ulcer prophylaxis	Prophylaxis with proton pump inhibitors for 4 wks.	None	Prophylactic proton pump inhibitors from acute to post-acute post-SCI timepoint.
**Moderate Priority**			
Airway & breathing	Intubate at first indication of respiratory dysfunction, ventilating with 10 mL/kg body mass & PEEP of 0-5 cmH_2_O.	Some laboratories intubate and ventilate animals during SCI surgery.	Measurement of post-injury blood O_2_ saturation.
Cardiovascular management	Correction of active bleeding, early infusion of I.V. fluids and/or blood, and norepinephrine administration to maintain MAP of 85-90 mmHg.	Fluid injections during and after surgery.	Monitor BP & HR in all post-SCI animals, and address bradycardia and hypotension with norepinephrine.
Nutrition	Early enteral feeding if swallow function allows, monitor/correct glycemia.	Diet supplementation & weight monitoring.	Assess aspiration risk prior to beginning feeding and monitor glycemia throughout recovery period.
Temperature regulation	Warm with blanketing & warmed fluids, adjust to prevent overheating.	Volitional access to heating pad.	Assess post-SCI body temperature & adjust temperature control appropriately.
**Low Priority**			
Anesthesia and spinal surgery	Closed reduction: Diazepam or meperidine Induction for surgery with general anesthesia: I.V. propofol, ketamine, and etomidate, not inhalables. Maintenance: For somatosensory evoked potentials I.V. propofol, inhaled sevoflurane, and I.V. opioid (remifentanil, sufentanil, or fentanyl) with any neuromuscular blocking agent. Motor evoked potentials (MEP) or electromyography (EMG) no neuromuscular blocking agents For MEP: propofol, sevoflurane, and an opioid For EMG: sevoflurane and an opioid Electroencephalogram or electrocorticography I.V. remifentanil and a muscle relaxant with either inhaled anesthetic (nitrous oxide or sevoflurane) or I.V. dexmedetomidine and propofol.	Most commonly: Xylazine (or medetomidine) cocktail with ketamine or isoflurane	Induction:Ketamine, etomidate, and propofol injection Maintenance (with neuromuscular blockers and mechanical ventilation) Continue propofol, add an opioid, inhalable isoflurane, and any neuromuscular blocker (succinylcholine).Mechanically ventilate. Maintenance (without neuromuscularblocker and ventilation):Continued propofol, add opioid, and isoflurane
**Unchanged**			
Antibiotics	Prophylaxis not indicated for SCI itself, but various antibiotics used for SCI complications and prophylaxis for surgery and penetrating trauma.			Either no prophylactic antibiotics or systemic prophylactic treatment with various agents.	No change.
Dermatologic care	Special bedding, positional shifting with frequent skin integrity inspection.	Surgical site care and regular assessment.		No change.
Urinary and bowel care	Urinary catheterization and bowel assistance as needed for prevention of bladder distension and ≥1 bowel movement per day.	Monitoring, bladder expression & bowel evacuation when needed.	No change.

### High priority aftercare considerations

#### Analgesics

Both clinical and veterinary care include interventions to manage pain through administration of analgesic drugs. Most animal protocols take a multi-modal approach, just as clinical standards recommend. The most glaring difference between clinical and experimental protocols, however, lies in the prevalent use of NSAID therapy in murine aftercare.

As discussed in our summary of clinical care, perioperative NSAID use is contraindicated and is also associated with bone growth impairment within the population of persons with acute SCI. In addition, a long list of common health conditions and prescribed medications contraindicate NSAID therapy. All of these reasons render acetaminophen a more appropriate non-narcotic analgesic than any NSAID.^19,30–32^

While some research groups have already utilized acetaminophen and an opioid to treat their animals, such practice remains uncommon.^84,87,116–19,121–124,126–128^ Thus, we strongly suggest the modification of murine aftercare protocols to include acetaminophen rather than carprofen or meloxicam along with buprenorphine. To speculate, it is possible that the common use of NSAIDs to date in experimental models of SCI could affect the acute inflammatory response, exacerbate peripheral organ dysfunction, and impair bone healing in a fashion not seen in the clinical setting, thus contributing to heterogeneity of outcome between bench and bedside interventions.

#### Anticoagulation

Anticoagulative therapy with subcutaneous LMWH is the clinical standard of care acutely after SCI because of the need for prevention of life-threatening coagulative complications such as pulmonary embolism that often arises from the immobility caused by SCI.^14,29^ No pre-clinical publication surveyed in this review included any anticoagulative therapy for post-SCI care. Most rats and mice, however, recover gross motor function quickly after SCI surgery and often are ambulatory within hours. This likely prevents hemostasis in murine animals, prevents thromboembolic complications from arising, and might explain the absence of anticoagulation from standard veterinary aftercare, although rodent studies to examine post-SCI coagulation are necessary to assert this with any confidence.

Regardless, we strongly suggest the addition of subcutaneous LMWH to murine treatment after experimental SCI to more accurately model the polypharmacy exposure of patients with clinical SCI. If consistent hemorrhage-related complications begin to arise, mechanical anticoagulative measures should be pursued as directed by clinical guidelines.

Few clinical care standards for SCI are as unequivocal as the anticoagulative measures recommending LMWH administration. Thus, the clinical SCI population is likely to have consistent anticoagulative exposure. It is vital to model this through pre-clinical animal studies of SCI, particularly in the context of blood–brain barrier disruption within the spinal cord and blood cell infiltration acutely after injury.

#### Stress ulcer prophylaxis

Clinical standards of care for prevention of stress ulcer formation strongly state that PPI therapy should be administered to all those with traumatic SCIs for four weeks. If counterindications to PPIs arise, such as *C. diff.* infection, histamine receptor blockers are used as an alternative. Stress ulcers, apart from detrimentally affecting quality of life through the pain they cause, often result in gastrointestinal bleeding and thus must be prevented in patients after SCI.

Although gastrointestinal ulceration can develop in response to stressful circumstances in murine animals, it is unknown whether C2Hx and high cervical contusion models of SCI cause stress ulcer formation in mice and rats.^141–143^ Even though it is likely that cervical SCIs constitute a severe enough stress to induce ulceration, it remains necessary to investigate the question through future experimentation.

Regardless, even if most hemisected and contused rodents do not form ulcers, inclusion of PPI therapy is valuable as a means of more closely modeling clinical gastric conditions and polydrug interactions involved with the majority of human SCI cases. Thus, we strongly suggest that prophylactic PPI therapy be included in veterinary aftercare protocols for mice and rats with experimentally induced SCI.

### Moderate priority aftercare considerations

#### Airway and breathing interventions

Although intubation and mechanical ventilation are often necessary to preserve life after cervical SCI in humans, the C2Hx and high cervical contusion models of SCI in rats and mice have been intentionally designed to induce a replicable injury and physiological deficit that preserves enough respiratory function for survival without prolonged intubation and mechanical ventilation. Those injury models have been so designed to diminish the cost of research and thus increase the experimental throughput to address the significant public health issue of respiratory dysfunction after cervical SCI. As a result, the clinical standards describing best practice for intubation and mechanical ventilation are not generally applicable to veterinary care strictly for C2Hx and contusion.

As a result, we do not advocate for any amendment to existing animal aftercare protocols on the basis of clinical intubation and mechanical ventilation. We included a summary of those standards for the sake of completeness and because of their general value for research concerning intubation and ventilator-associated sequalae or respiratory function post-SCI.

If the field of murine cervical SCI were to standardize oximetric monitoring of O2 saturation in the acute post-SCI setting, respiratory deficits induced by experimental injuries between various laboratories might be better defined. Further, if animal subjects fail to maintain O2 saturation on room air, supplemental oxygen should be administered to parallel clinical treatments to prevent persistent hypoxemia. The price and other obstacles associated with initializing the use of pulse oximeters for mice and rice after C2Hx and contusion surgeries could put undue strain on resources for some research groups and so we suggest acute post-SCI oximetry only as a moderate-priority modification to current veterinary aftercare protocols.

#### Cardiovascular management

Both veterinary and clinical protocols include fluid resuscitation to replenish blood volume loss occurring as a result of SCI trauma. None of the animal aftercare surveyed in this review, however, contains BP or HR monitoring and no hemodynamic treatment was administered through vasopressor or chronotropic drugs. Clinically, BP and heart rate drugs are not implemented prophylactically, but rather in response to deficits in cardiovascular function detected through real-time monitoring.

To emphasize the importance of post-SCI cardiovascular management, recent analysis of historical SCI data indicates that perioperative BP is a major predictor of overall health and neuromotor outcome.^144,145^ Thus, for the short term, we suggest that veterinary aftercare measures be modified to include BP and HR monitoring acutely post-surgery and treatment instituted according to clinical guidelines to correct both hypotension and bradycardia while avoiding hypertension.

#### Nutrition

For clinical care of persons with SCI, enteral feeding should be begun as soon as possible once risk of aspiration is evaluated. Parenteral feeding is only to be used if serious enough aspiration risk or gastrointestinal complications prevent feeding by mouth. In the veterinary setting after C2Hx and contusion, subjects are usually provided full access to food immediately after return to their home cages and often given nutritional supplements, fruit, or other salient food choices. Neither aspiration risk nor blood glucose level is standardly assessed in post-SCI murine animals, however. Aspiration of food matter into the airways, apart from diminishing the efficiency of feeding, can promote respiratory infection and impede gas exchange within the lung. As discussed above, hyperglycemia can lead to increased death and disability while hypoglycemia indicates ineffective nutrition as well as worsens neurological outcome.

Thus, it is important to assess for these concerns to recognize and tailor feeding strategies accordingly in a more clinically relevant and effective fashion. Our suggestion for these nutrition-based amendments to veterinary protocols are only moderate in strength because of the demanding nature of the resources required to assess aspiration risk. Aspiration assessment requires radiographic imaging capabilities and thus might be untenable for laboratories not already equipped. In comparison, blood glucose assessment most likely would be simpler to enact because glycemia assays require only small sample volumes and protocols are easily doable for most biomedical research laboratories.

#### Temperature regulation

Body temperature is not usually monitored in rats and mice once the SCI-inducing surgery is complete, but subjects typically recover with access to heating pads. In the clinic, body temperature is closely monitored, and both hypothermia and hyperthermia are detected and resolved as they arise. Thus, we suggest the addition of frequent measurement of animals' body temperature to aftercare protocols after experimentally induced SCI in rats and mice. This will allow experimenters to adjust temperature control settings to maintain adequate body temperature.

### Low priority aftercare considerations

#### Anesthesia and spinal surgery

The spinal surgeries described in our clinical summary are intended to decompress neural tissue, correct instability of the spine, or remove embedded foreign objects, thus preserving function and preventing exacerbation of damage from spontaneously sustained SCIs. In contrast, the mouse and rat surgeries we discussed are used to induce the SCI, rather than manage it. Intuitively, the indications for clinical intervention with surgery would not apply to aftercare for experimentally induced SCI in mice and rats. The anesthesia protocols used in the clinic, however, might be used as a guide for modifications to veterinary anesthesia for C2Hx and contusion surgeries.

No experimental SCI models discussed in this review necessitate intraoperative neuromonitoring, and so we suggest modeling anesthetic induction and maintenance after the least restrictive elements described by the summary of clinical protocols in Table 1. The clinical induction of anesthesia would consist of multiple agents, specifically intravenous propofol, ketamine, and etomidate. Without respect to neuromonitoring, maintenance of anesthesia would consist of intravenous propofol and an opioid, coupled with an inhaled anesthetic (sevoflurane or isoflurane), and a neuromuscular blocker.

Etomidate and propofol are both foreign to the anesthetic protocols used for murine SCI surgeries described in this review, but the N-methyl-d-aspartate (NMDA) receptor blocker ketamine is already commonly used for both induction and maintenance.^146^ The primary mechanism of action for both etomidate and propofol consists of positive modulation of the inhibitory gamma-aminobutyric acid (GABA) system, which is distinct from the effects of the alpha-2 adrenoreceptor agonists xylazine or medetomidine that are commonly paired with ketamine for veterinary anesthesia.^147–150^ Thus, a modified veterinary protocol for anesthesia induction would consist of ketamine with GABA agonists instead of xylazine.

Although some laboratories ventilate their animals during C2Hx or high cervical contusion surgeries, many do not, because it is usually unnecessary for subject survival.^52–64^ As part of general anesthesia, systemic neuromuscular blockers paralyze not only skeletal muscles, but respiratory musculature as well. Thus, intubation and mechanical ventilation are required during anesthesia with such drugs. For the most clinically comparable anesthetic maintenance protocol for experimental SCI, subjects would continue on intravenous propofol and would add an opioid with an inhalable agent such as sevoflurane or isoflurane. In addition, animals would receive a neuromuscular blocker like succinylcholine and would all be intubated and ventilated.

Such a demanding anesthetic protocol, however, would increase the resources required for each experiment to a degree that could significantly constrain productivity for many laboratories and inhibit rather than enhance translation of pre-clinical therapeutics to clinical care. A more practical modification of anesthesia maintenance would consist of induction with GABA agonists and ketamine, followed by maintenance with continued GABA agonists along with an opioid, and inhaled isoflurane without any neuromuscular blockade or need for mechanical ventilation.

Most SCI-inducing surgeries in murine animals are significantly shorter in duration and less complicated than clinical spinal procedures. Thus, the duration of experimental subjects' exposure to anesthetic drugs is much less than in clinical cases. For this reason, the specific protocol used for anesthesia during experimental SCI surgeries is likely less significant than therapeutic measures employed after injury. Recognizing that researchers have limited time and resources, we suggest that laboratories prioritize modifications to post-SCI aftercare rather than first attempting to conform their anesthesia protocol to clinical standards.

### Aftercare with no considered changes

#### Antibiotics

Prophylactic treatment with antibiotics is not indicated for acute SCI alone according to clinical standards of care.^13^ Prophylaxis is recommended, however, in cases wherein spinal surgery is necessary and when there is polytrauma involving open fractures or penetrating thoracic wounds.^13^ After experimentally induced SCI in mice and rats, some laboratories initiate prophylactic antibiotic therapy and others do not. Responsive antibiotic treatment of complications that arise after SCI in the clinical setting often include respiratory and UTIs, but after experimentally induced SCI in mice and rats, obvious signs of infection are unusual and subjects displaying any unusual medical complications in the post-operative stage are usually excluded from the study.^28^ As a result, implementation of responsive antibiotic therapy for post-SCI rats and mice would be unnecessary in most cases.

Because all experimental animals in SCI studies undergo surgery to induce their SCI, it could be argued that prophylactic antibiotic treatment is merited according to clinical standards. There is no homogenous protocol for the specific antibiotic combination utilized for post-surgical prophylaxis, however, because such details are highly dependent on local prevalence of antibiotic-resistant bacterial strains and both patient allergies and comorbidities affecting drug metabolism and toxicity. Overall, clinical guidelines for infection treatment and prophylaxis after SCI result in a high degree of variation in patient exposure to antibiotics such that even patients in the same hospital might be treated with distinct medications and other persons with SCI receive no antibiotics at all.

As a result, we suggest no change in individual research groups' antibiotic treatment protocol with respect to infection prophylaxis. The heterogeneity of antibiotic type and exposure currently present in experimental studies of SCI already seems to reflect the clinical variation of antibiotic treatment in the human population. Regardless, it remains of great importance to report all post-injury drug treatment used in pre-clinical studies and to conduct replications of experiments evaluating therapeutic strategies across various profiles of antibiotic exposure to more robustly support translation of promising treatments to the clinic.

#### Dermatological care

As discussed briefly in the anticoagulation section above, mice and rats usually have greater mobility after SCI than do spontaneously injured humans. As a result, animals are much less prone to the development of pressure sores. Veterinary aftercare already includes frequent monitoring of skin integrity, specifically at the surgical site and areas such as the nares, eyes, and mouth prone to porphyrin staining. The clinical protocols dedicated to dermatological care primarily describe techniques to shift the weight-bearing skin surface and considerations to keep dependent areas clean and dry to avoid breakdown. At the current stage, we suggest no additional modifications to veterinary standards of skin care to reflect the clinical setting.

#### Urinary and bowel care

In both clinical standards of care for acute SCI and pre-clinical animal models of high cervical SCI, urinary and bowel dysfunction are typically monitored and addressed responsively, rather than prophylactically. In post-SCI mice and rats, urination is facilitated through manual voiding rather than the more clinical approach of catheterization. Techniques for facilitation of bowel movements are not as clearly standardized in clinical standards regarding cervical SCI, and most animal care consists of manual bowel evacuation.

Overall, urinary and bowel care protocols are comparable between current clinical care and most veterinary studies. Although bladder catheterization would be a more clinically comparable urinary care intervention than manual voiding, any added clinical relevance (e.g., potential increased risk of UTI) the intervention is unlikely to justify the additional cost, time, and technical skill necessary to implement such a change. As a result, we do not suggest any change to commonly utilized bladder and bowel care protocol for post-SCI mice and rats.

## Conclusion

More than half of all spinal cord injuries occur at the cervical level and often lead to life-threatening breathing motor dysfunction.^1^ The C2 hemisection and high cervical contusion mouse and rat models of SCI are widely utilized both to understand the pathological effects of SCI and to develop and evaluate potential therapies. Despite rigorous efforts, no pre-clinical therapeutic has yet become part of the clinical standard of care.

For the first time, in this review we have analyzed the similarities and differences between medical treatment administered to persons with SCI and veterinary care for experimental animals. Through this effort, we have identified areas of marked dissimilarity that could differentially interact with experimental therapeutics, influencing the heterogeneity of outcome between pre-clinical and clinical studies. To remedy this, we proposed several modifications to veterinary aftercare protocols for SCI to render them more representative of clinical care (Table 3).

- Considered changes of high priority are listed below:
- Carry out analgesic therapy with a combination of acetaminophen and buprenorphine and exclude NSAID use from traumatic post-SCI aftercare.
- Institute prophylactic anticoagulation for all experimental subjects with administration of subcutaneous LMWH acutely after SCI until chronic stages.
- Institute prophylactic therapy to prevent stress ulcer formation by treating all animals with a PPI from acute to post-acute post-injury time points.
- Other suggestions of more moderate priority are as follows:
- Institution of O2 saturation monitoring for prevention of prolonged or chronic hypoxemia and enhanced characterization of respiratory deficits after hemisection and contusion injuries.
- Employ blood pressure and heart rate monitoring after experimental SCI to ensure that standard fluid administration is sufficient to prevent hypotension and other cardiovascular dysfunction, instituting vasoactive and cardioactive drugs as needed.
- Assess aspiration risk and analyze blood glucose levels before and during feeding for safer, more effective nutritional support.
- Assess each post-operative mouse and rat's body temperature at frequent intervals during recovery to adjust temperature control and prevent both hypothermia and hyperthermia.
- A lower priority modification includes:
- Adjustment of protocols used to anesthetize mice and rats for SCI surgeries.

An important facet of our findings lies within the significant heterogeneity of animal care protocols between (and even within) different research groups and laboratories participating in cervical SCI research. One of the goals of this review is to encourage the unification of veterinary care protocols between laboratories by modeling each care intervention after a clinical standard.

It is important to recognize that the heterogeneity of the clinical population of persons with SCI will never perfectly be recapitulated by any experimental model of injury. The identification and amendment of inconsistencies between experimental and clinical protocols as we suggest, however, will address some of the obstacles constraining translation of therapies from the bench to bedside. Although this effort will take time, we predict that it will result in more clinically relevant experimental models of SCI that can be more effectively used to evaluate therapies and eventually bridge the translational “Valley of Death” to ultimately restore independent breathing function to persons with cervical SCI.

## Transparency, Rigor, and Reproducibility Summary

No new data was generated for this review. Thus, no human or animal subjects were used. A complete list of reviewed and referenced works can be found in our Works Cited section below and we completely described our methodology for finding sources and completing our literature search in our methods section. Furthermore, the authors agree to be available for contact by parties interested in discussing this review and its methodology further.
